# Supplementary material for: Optimization of the Growth Process of Double Perovskite Pr2−δNi1−xMn1+xO6−y Epitaxial Thin Films by RF Sputtering
Source: Materials (Basel). 2022 Jul 20;15(14):5046. doi: 10.3390/ma15145046 (PMC9324673; doi:10.3390/ma15145046)
Supplement: Supplementary file 1 [file materials-15-05046-s001.zip › materials-1802593-supplementary.pdf]

SUPPORTING INFORMATION

Article

Optimization of the Growth Process of Double Perovskite $Pr_{2-\delta}Ni_{1-x}Mn_{1+x}O_{6-y}$ Epitaxial Thin Films by RF Sputtering

Monica Bernal-Salamanca [1,*], Lluis Balcells [1], Zorica Konstantinović [2], Alberto Pomar [1], Benjamín Martínez [1] and Carlos Frontera [1]

[1] Institut de Ciència de Materials de Barcelona, Consejo Superior de Investigaciones Cinetíficas (ICMAB-CSIC), Campus UAB, 08193 Bellaterra, Spain; balcells@icmab.es (L.B.); apomar@icmab.es (A.P.); bejamin.martinez@icmab.es (B.M.); frontera@icmab.es (C.F.)

[2] Center for Solid State Physics and New Materials, Institute of Physics Belgrade, University of Belgrade, Pregrevica 68, 6080 Belgrade, Serbia; zorica@ipb.ac.rs

* Correspondence: monakob24@gmail.com

1. XRD pattern of the final target of Pr_2NiMnO_6

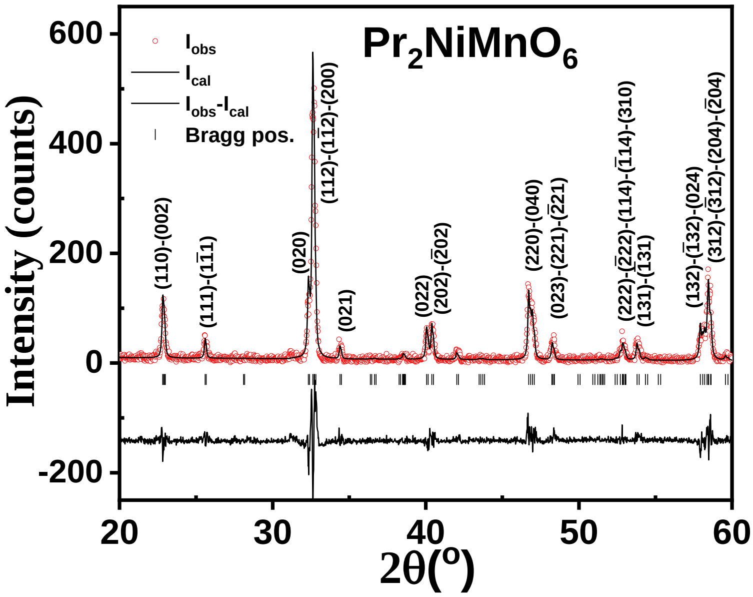

Figure S1. XRD pattern of the final target of Pr_2NiMnO_6 prepared by solid state reaction method. The peaks clearly show the primary contribution of the PNMO phase. I_{obs}, I_{cal} represent XRD of the observed and calculated intensities, respectively, and (|) the Bragg position.

Optimization of growth conditions

1. Oxygen pressure deposition

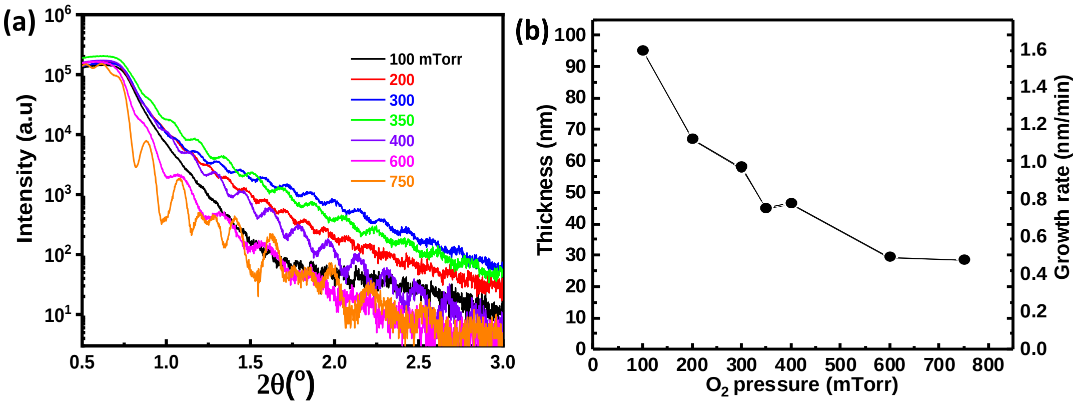

Figure S2. (**a**) XRR data of PNMO thin films on STO substrates grown at 850°C under different oxygen pressures ranging from 100 to 750 mTorr. (**b**) Thickness and growth rate as a function of oxygen pressure of the same samples.

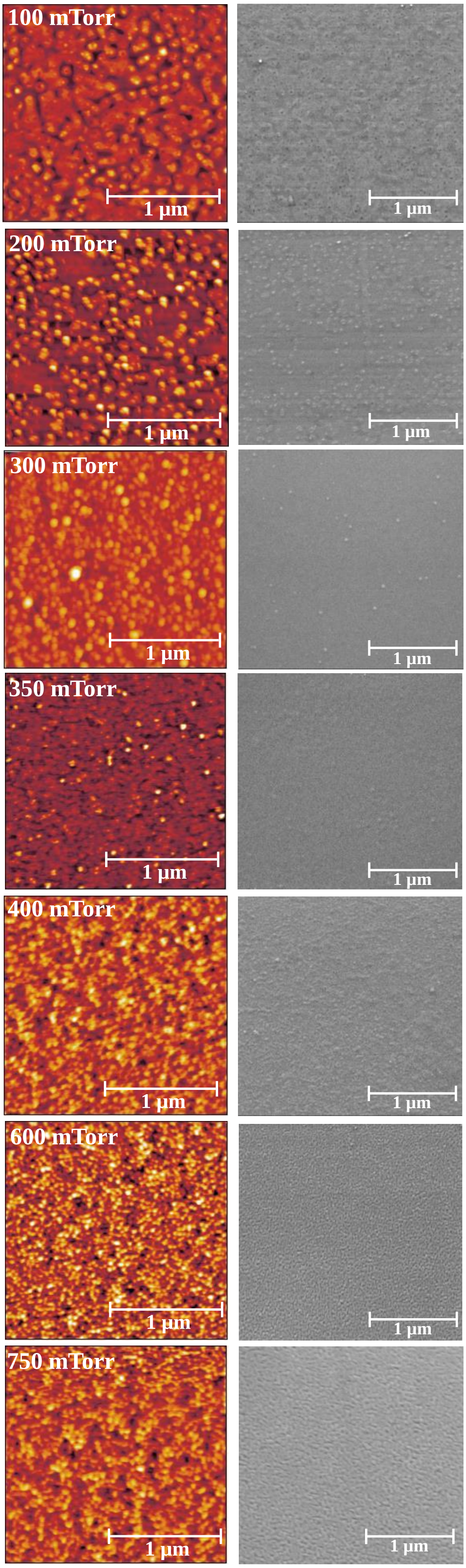

Figure S3. AFM topography (2x2 µm^2 area, left-hand image) and SEM micrographs of corresponding AFM images (right-hand image) of PNMO thin films grown under different oxygen pressures (ranging from 100 to 750 mTorr) at 850°C, with in-situ annealing at the same growth temperature (1h under 420 Torr O_2.).

2. Temperature deposition

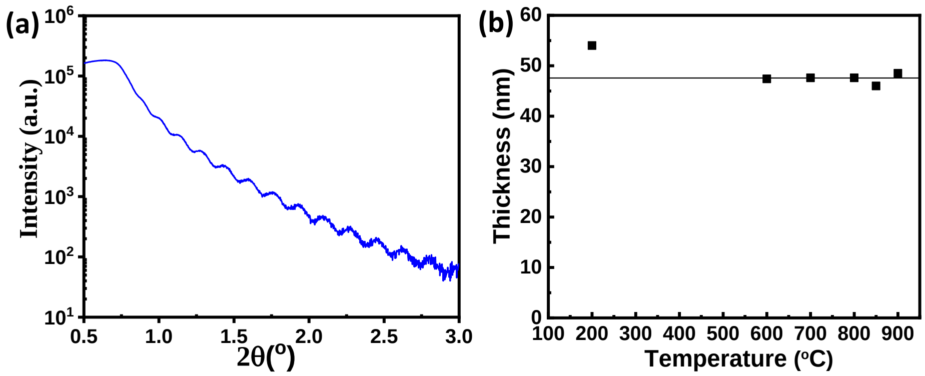

Figure S4. (**a**) XRR data of a PNMO film deposited at a temperature of 700°C under 350 mTorr O_2. (**b**) Thickness as a function of growth temperature.

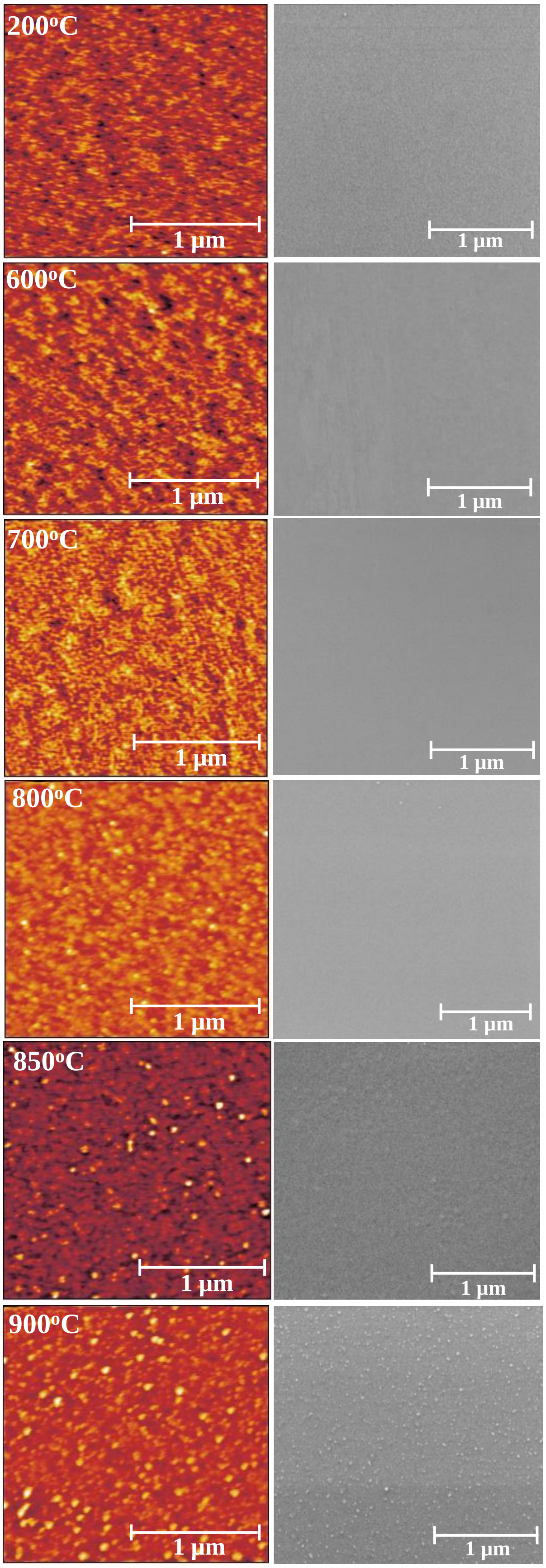

Figure S5. AFM topography (2 × 2 µm[2] area, left-hand images) and SEM micrographs of corresponding AFM images (right-hand images) of PNMO thin films grown under 350 mTorr O_2 at different growth temperatures (ranging from 200ºC to 900ºC) with in-situ annealing at the same growth temperature (1h under 420 Torr O_2.).
